# Clinical Characteristics and Treatment Outcomes of Thyroid Cancer at a Tertiary Care Hospital in Najran Region, Saudi Arabia: A Single-Centre Retrospective Study

**DOI:** 10.7759/cureus.72380

**Published:** 2024-10-25

**Authors:** Ahmed M Badheeb, Samer Alkarak, Mana A Alhajlan, Rakan Alwadai, Ali M Al-Qannass, Abbas H Almakrami, Abdelaziz A Aman, Hossam A Hussein, Nadeem M Nagi, Mohammed A Fagihi, Islam A Seada, Ahmed Harwn, Saleh M Alqahtani, Ibrahim Mokhtar, Abdullah Abu Bakar, Faisal Ahmed, Mohamed Badheeb

**Affiliations:** 1 Oncology, King Khalid Hospital, Oncology Center, Najran, SAU; 2 Medicine, Hadhramaut University, Mukalla, YEM; 3 General Surgery, King Khalid Hospital, Najran, SAU; 4 Otolaryngology - Head and Neck Surgery, Armed Forces Hospital Southern Region, Khamis Mushait, SAU; 5 Endocrinology, King Khalid Hospital, Najran, SAU; 6 Internal Medicine/Endocrine and Diabetes, King Khalid Hospital, Najran, SAU; 7 Otolaryngology - Head and Neck Surgery, Menoufiya University, Shebien Elkoom, EGY; 8 Otorhinolaryngology, King Khalid Hospital, Najran, SAU; 9 Oncology, King Khalid Hospital, Najran, SAU; 10 Surgical Oncology, King Khalid Hospital, Najran, SAU; 11 Cardiothoracic Surgery, King Khalid Hospital, Najran, SAU; 12 Nuclear Medicine/Radiology, King Khalid Hospital, Najran, SAU; 13 Internal Medicine, King Khalid Hospital, Najran, SAU; 14 Ophthalmology, King Khalid Hospital, Najran, SAU; 15 Urology, Ibb University, Ibb, YEM; 16 Internal Medicine, Yale New Haven Health, Bridgeport Hospital, Bridgeport, USA

**Keywords:** cancer, mortality, najran region, saudi arabia, survival, thyroid, treatment outcome

## Abstract

Background

The global incidence of thyroid cancer has increased significantly over the past decades. This study aims to review the clinical characteristics and treatment outcomes of thyroid cancer at the Tertiary Care Hospital in the Najran region of Saudi Arabia.

Material and methods

We conducted a retrospective study of 279 patients diagnosed with thyroid cancer at our hospital from March 2014 to December 2021. Clinicopathologic parameters were obtained from the patient's medical records and examined using univariate and multivariate Cox regression to identify independent predictive markers.

Result

The mean age was 42.8 ±14.5 years, and most cases were female (n= 203, 72.8%). Most cases (n=170, 60.9%) underwent total thyroidectomy. Additionally, lymph node dissection was performed in 28 (10.0%) cases. Localized disease, distant, and regional metastasis were observed in 214 (76.7%), 34 (12.2%), and 31 (11.1%), respectively. The neck lymph nodes and lungs were the most common metastasis regions in 19 (6.8%) and 11 (3.9%) cases, respectively. Papillary thyroid cancer and follicular thyroid cancers accounted for the majority of cases in 236 (84.6%) and 33 (11.8%), respectively. Adjuvant therapy, including radioactive iodine ablation, was reported in 51 (18.3%) and external beam radiotherapy in four (1.4%). Independent prognostic factors of overall mortality of thyroid carcinoma were older age (Hazard ratio (HR):1.05, 95% confidence interval (CI): 1.01-1.09, p=0.008), Diabetic mellitus (HR: 4.30, 95% CI: 1.11-16.62, p=0.035), pathologic subtype of follicular carcinoma (HR: 4.48, 95% CI: 1.07-18.73, p=0.040) or non-papillary thyroid carcinoma subtypes (HR: 12.56, 95% CI: 2.44-64.74, p=0.002), metastasis presentation (HR: 11.70, 95% CI: 3.30-41.46, p<0.001), pulmonary metastasis (HR: 27.92, 95% CI: 6.96-111.98, p<0.001), bone and liver metastasis (HR: 15.20, 95% CI: 1.70-135.98, p=0.015), tumor size >4 cm (HR:121.21, 95% CI: 15.33-958.34, p<0.001), and extrathyroidal extension (HR: 6.15, 95% CI: 1.59-23.77, p=0.009).

Conclusion

This study demonstrates that advanced age, the presence of diabetes, non-papillary thyroid carcinoma subtypes, metastatic disease, tumor size greater than 4 cm, and extrathyroidal extension are independently associated with a poorer prognosis in patients with thyroid carcinoma. To offer the finest modern care, a multidisciplinary approach should be employed when developing a tailored treatment strategy, considering relevant recommendations and stratification systems.

## Introduction

The increased use of imaging modalities, followed by subsequent invasive testing, has led to a rise in the incidence and prevalence of thyroid cancer globally [1,2]. Epidemiological studies report a higher incidence of thyroid cancer in high-income regions, including North America and Europe. However, high rates have also been reported in Asian regions, such as China [3]. In the United States, thyroid carcinoma is the 13th most prevalent cancer overall and the sixth most common among women [4]. These figures differ from those in the Kingdom of Saudi Arabia (KSA), where recent reports show thyroid cancer as the second leading malignancy among females, with a 26-fold increase from 1990 to 2016 [5]. More recent data from the Saudi Cancer Registry indicate that thyroid cancer is the third most common malignancy among Saudi adults and the leading malignancy among younger females aged 15-29 years [6]. 

While the increased incidence may be partly attributed to the frequent use of imaging, improved accessibility to healthcare services and increased social awareness are likely contributing factors [7]. Notably, there is a significant variation in cancer distribution across different regions of KSA. In the Najran region, thyroid cancer accounts for the majority of cancer cases (15.8%), contrasting with other regions where breast, colorectal, and hematological malignancies predominate [6]. There is a notable scarcity of literature regarding the patterns of thyroid malignancies, patient profiles, and predictors of outcomes. This study aims to examine the clinical and histological characteristics of differentiated and poorly differentiated thyroid cancer, as well as the factors associated with survival over nine years in an oncology referral hospital in Najran, Saudi Arabia.

## Materials and methods

Study design

A retrospective chart review study covered the period between March 1, 2014, to December 31, 2021, involving 279 adult patients with thyroid nodules who were presented to King Khaled Hospital, Najran, Saudi Arabia. This study was approved by the Ethics Research Committees of Najran Health Directorate (Code: KACST, KSA: H-I1-N-089) in compliance with the ethical standards outlined in the Declaration of Helsinki. Owing to the study's retrospective nature, written informed consent from the included patients was waived. Patients less than 18 years old and patients without confirmed thyroid cancer diagnosis were excluded.

Collected data

The patients' electronic medical records were reviewed, and the extracted data included patients’ demographics, comorbidities, family history of thyroid cancer, initial presentation, and ultrasonographic findings with corresponding Thyroid Imaging Reporting and Data System (TI-RADS™) scores. Additionally, pathological findings from fine-needle aspiration cytology (FNAC), based on the Bethesda System for Reporting Thyroid Cytopathology (TBSRTC), were obtained, along with operative approaches, findings, and surgical staging. Post-operative data included complications, thyroglobulin levels, radioiodine scan (RAI) findings, and overall patient outcomes. Data were collected through independent chart reviews. The collected data were thoroughly assessed for accuracy, completeness, and consistency. In cases where contradictory or missing information was identified, the charts were reviewed and reevaluated to ensure data quality.

Ultrasonography (USG) findings

Thyroid ultrasounds were conducted by qualified radiologists using the TI-RADS and American College of Radiology (ACR) 2017 criteria [8]. The nodule composition, echogenicity, form, margin, and echogenic foci were evaluated. Each feature was evaluated using ACR TI-RADS, with scores ranging from 0 to 2 for composition, 3 for echogenicity, form, and margin, and 3 for echogenic foci [9].

FNAC findings

The FNAC was performed under USG guidance only. FNA was done on the thyroid nodules, and the cytological diagnosis was determined using Bethesda's international cytological classification. Cytopathology reports were categorized into six types: Bethesda I, II, III, IV, V, and VI represent unsatisfactory material, benign, atypical/follicular lesion, suspected follicular neoplasia, suspected malignancy, and malignancy, respectively [10]. Overall survival (OS) was calculated from diagnosis to death.

Clinical assessment and follow-up

Lobectomy and isthmectomy were executed for T1 and T2 tumors localized to unilateral lobes. At the same time, total thyroidectomy was indicated for T3 and T4 tumors or in patients exhibiting high-risk factors such as multifocality, lymphatic or distant metastasis, familial predisposition, and prior ionizing radiation exposure. In certain instances where postoperative radionuclide therapy was anticipated, total thyroidectomy was deemed appropriate. Central neck dissection was carried out for cN1 and the majority of cN0 patients, with modified lateral lymph node dissection, applied to those with clinically suspicious lateral metastases. Pathological confirmation was obtained for all samples, and thyrotrophic (Thyroid Stimulating Hormone) suppressive therapy was the primary postoperative treatment, complemented by radioactive iodine for advanced PTC cases. Follow-up protocols included routine assessments via neck palpation, ultrasound, and thyroid function tests, with imaging modalities like computed tomography (CT)/magnetic resonance imaging (MRI) and needle biopsy reserved for suspected recurrences or metastases. At the same time, elevated thyroglobulin levels without identified lesions post-surgery did not constitute adverse events for disease-free survival analysis. Local recurrence, distant metastasis, or death were unfavorable outcomes in the disease-free survival (DFS) study. The diagnosis of local recurrence should be validated by pathology. The 1-year and 5-year survival periods were defined as being alive for 365 and 1825 days after diagnosis, respectively. It should be noted that the 1-year relative survival estimates are more current than the 5-year survival statistics due to the survival technique used. We used the eighth edition of the American Joint Committee on Cancer/tumor node metastasis (AJCC/TNM) staging system to predict disease-specific mortality and the American Thyroid Association (ATA) risk stratification system to predict the risk of recurrent or persistent disease.

Primary outcome

The primary outcome was to report the clinical, radiological, and histological characteristics of thyroid cancer, treatment, and overall survival. The secondary outcome was documenting the factors associated with survival in thyroid cancer patients.

Statistical analysis

We utilized the mean ± standard deviation (SD) to represent the quantitative variables, and the frequency (percentage) was employed to describe the qualitative variables. Chi-squared tests were used to compare the characteristics of patients and tumors. Kaplan-Meier survival curves and Cox-proportional hazard methods were applied for survival analyses. The relationship between pre-therapeutic variables and overall survival was reported as a hazard ratio (HR) with a 95% confidence interval (CI). A P-value less than 0.05 was deemed statistically significant. All the data were processed using the SPSS version 20 software (IBM Corp., Armonk, USA).

## Results

Participants' demographic and baseline characteristics

The mean age was 42.8 ±14.5 years (Range 19- 89 years), most cases were aged between 30-39 (n= 85, 31%) followed by between 40-49 (n= 73, 26%), and most cases were female (n= 203, 72.8%). Comorbidities include hypertension, diabetic mellites, history of thyroid cancer, and cerebrovascular accidents in 33 (11.8%), 26 (9.3%), 11 (3.9%), and four (1.4%), respectively. The most commonly reported symptom was dysphagia (n=266, 95.3%), and most cases were diagnosed at least three months after the presenting symptom (n=176, 63.5%). The TIRADS showed a malignant feature in most cases and were presented as TIRADS 5 in 117 (41.9%) of cases, while TIRADS 1 and TIRADS 2 were mentioned in 7 (2.5%) and 19 (6.8%), respectively. Most cases were reported initially as malignant according to FNA results based on the Bethesda System and categorized as Category 6, Category 5, and Category 4 in 133 (47.7%), 105 (37.6%), and 24 (8.6%), respectively. However, 14 (5.0%) were reported as benign nodules (Category 2), one (0.4%) was reported as nondiagnostic or unsatisfactory (Category 1), and two (0.7%) were reported as atypia or follicular lesions of undetermined significance (Category 3) (Table 1).

**Table 1:** Clinicopathological profile of thyroid disease cases (N= 279) Abbreviation: TIRADS, Thyroid Imaging Reporting and Data System; FNA, fine-needle aspiration; SD, standard deviations.

Variables	N (%)
Age (year), Mean ±SD	42.8 ±14.5
Age group	
Less than 19 years	5 (1.8%)
Between 20–29 years	40 (14%)
Between 30–39 years	85 (31%)
Between 40–49 years	73 (26%)
Between 50–59 years	38 (14%)
Between 60–69 years	20 (7.2%)
Between 70–79 years	9 (3.2%)
More than 80 years	8 (2.9%)
Gender	
Male	76 (27.2%)
Female	203 (72.8%)
Comorbidity	
Hypertension	33 (11.8%)
Diabetic mellites	26 (9.3%)
History of thyroid cancer	11 (3.9%)
Cerebrovascular accidents	4 (1.4%)
Main symptoms	
Dysphagia	266 (95.3%)
Goiter	248 (88.9%)
Pain	164 (58.8%)
Dyspnea	79 (28.3%)
Hoarseness	23 (8.2%)
Cervical lymphadenopathy	19 (6.8%)
The time between the first symptom and diagnosis	
< 3 months	176 (63.5%)
Between 3-6 months	28 (10.1%)
Between 6-12 months	5 (1.8%)
Between 12-24 months	9 (3.2%)
> 24 months	15 (5.3%)
Not mentioned	46 (16.6%)
TRIAD	
TIRADS 1: normal thyroid gland	7 (2.5%)
TIRADS 2: benign nodules	19 (6.8%)
TIRADS 3: mildly suspicious of malignancy	68 (24.4%)
TIRADS 4: moderately suspicious of malignancy	68 (24.4%)
TIRADS 5: highly suspicious of malignancy	117 (41.9%)
FNA result based on Bethesda System	
Category 1: nondiagnostic or unsatisfactory	1 (0.4%)
Category 2: benign	14 (5.0%)
Category 3: atypia, follicular lesion of undetermined significance	2 (0.7%)
Category 4: Follicular neoplasm or suspicious for follicular neoplasm	24 (8.6%)
Category 5: suspicious for malignancy	105 (37.6%)
Category 6: Malignant	133 (47.7%)

Operative and postoperative characteristics

Most cases (n=170, 60.9%) underwent total thyroidectomy, followed by near-total thyroidectomy and lobectomy in 96 (34.4%) and 9 (3.2%), respectively. Additionally, lymph node dissection was performed in 28 (10.0%) cases. In most cases, the disease was localized (n=214, 76.7%). Meanwhile, distant and regional metastasis were reported in 34 (12.2%) and 31 (11.1%), respectively. The commonly reported metastasis locations were neck lymph nodes and lungs in 19 (6.8%) and 11 (3.9%) cases, respectively. The tumor was more significant than 40 mm in 22 (7.9%), multifocal in 2 (0.7%), and extra thyroid invasion in 19 (6.8%) (Table 2).

**Table 2:** Types of operative treatment and pathologic features

Variables	N (%)
Surgical procedure	
Total thyroidectomy	170 (60.9%)
Near-total thyroidectomy	96 (34.4%)
Total thyroidectomy with lymph node dissection	28 (10.0%)
Lobectomy	9 (3.2%)
Biopsy or no surgery	4 (1.4%)
Stage	
Localized	214 (76.7%)
Regional	31 (11.1%)
Distant metastasis	34 (12.2%)
Metastasis location	
Neck lymph nodes ­­­­	19 (6.8%)
Lung	11 (3.9%)
Bone	2 (0.7%)
Liver	2 (0.7%)
Tumor pathologic features	
Primary tumor size > 40 mm	22 (7.9%)
Multifocality	2 (0.7%)
locally advanced	4 (1.4%)

Pathologic and follow-up characteristics

The most commonly final histopathology report was Papillary thyroid carcinoma in 236 (84.6%), followed by follicular thyroid carcinoma in 33 (11.8%). Other reports were anaplastic carcinoma of the thyroid, medullary thyroid carcinoma, Well-differentiated thyroid tumor of uncertain malignant potential, Hurthle cell cancer, and non-Hodgkin's lymphoma in four (1.4%), one (0.4%), two (0.7%), and two (0.7%), respectively. Following surgery, adjuvant therapy, including radioactive iodine (RAI) ablation, was reported in 51 (18.3%) and external beam radiotherapy in four (1.4%) (Table 3).

**Table 3:** Final histopathology and follow-up report. Abbreviations: SD, standard deviations.

Variables	N (%)
Final histopathology report	
Papillary thyroid carcinoma	236 (84.6%)
Follicular thyroid carcinoma	33 (11.8%)
Well-differentiated thyroid tumor of uncertain malignant potential	1 (0.4%)
Medullary thyroid carcinoma	1 (0.4%)
Anaplastic carcinoma of the thyroid	4 (1.4%)
Hurthle cell cancer	2 (0.7%)
Non-Hodgkin's lymphoma	2 (0.7%)
Postoperative evolution	
Relapse or recurrence	15 (5.4%)
Residual mass in thyroid scan	40 (14.3%)
Adjuvant therapy	
Radioactive iodine ablation	51 (18.3%)
External beam radiotherapy	4 (1.4%)
Follow-up time (months), Mean ±SD	113.1 ±24.2
Status	
Alive	269 (96.4%)
Dead	10 (3.6%)

Survival analysis

**Figure 1:**
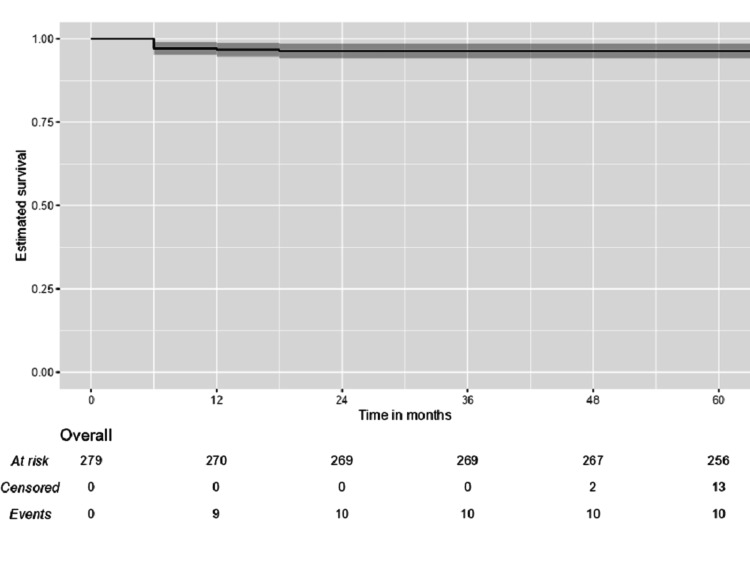
Kaplan-Meier survival curves for overall survival among thyroid cancer patients.

Within the follow-up period, residual mass in the thyroid scan was detected in 40 (14.3%), and relapse or recurrence occurred in 15 (5.4%). Ten (3.6%) died during the follow-up period. The median overall survival was 120 months. The one- and five-year survival was 96.77% (95% CI: 94.72%-98.87%) and 96.42% (95% CI: 94.26%-98.62%), respectively (Figure 1).

Factors associated with mortality in thyroid carcinoma

**Figure 2:**
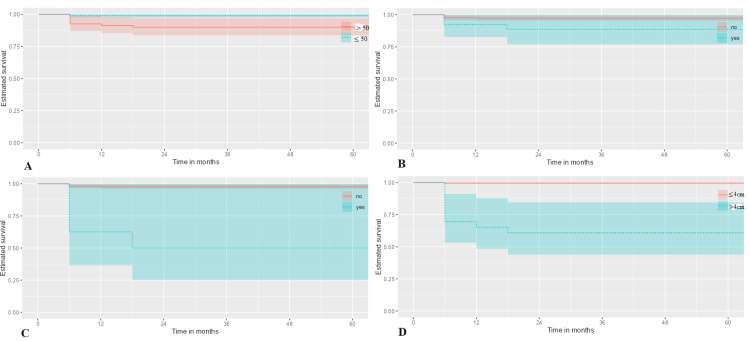
Among thyroid cancer patients, cox-proportional hazard methods found a statistically significant association between mortality and (A) older age (p=0.008), (B) diabetic mellitus (p=0.035), (C) extrathyroidal extension (p=0.009), and (D) tumor size larger than 4 cm (p<0.001).

**Figure 3:**
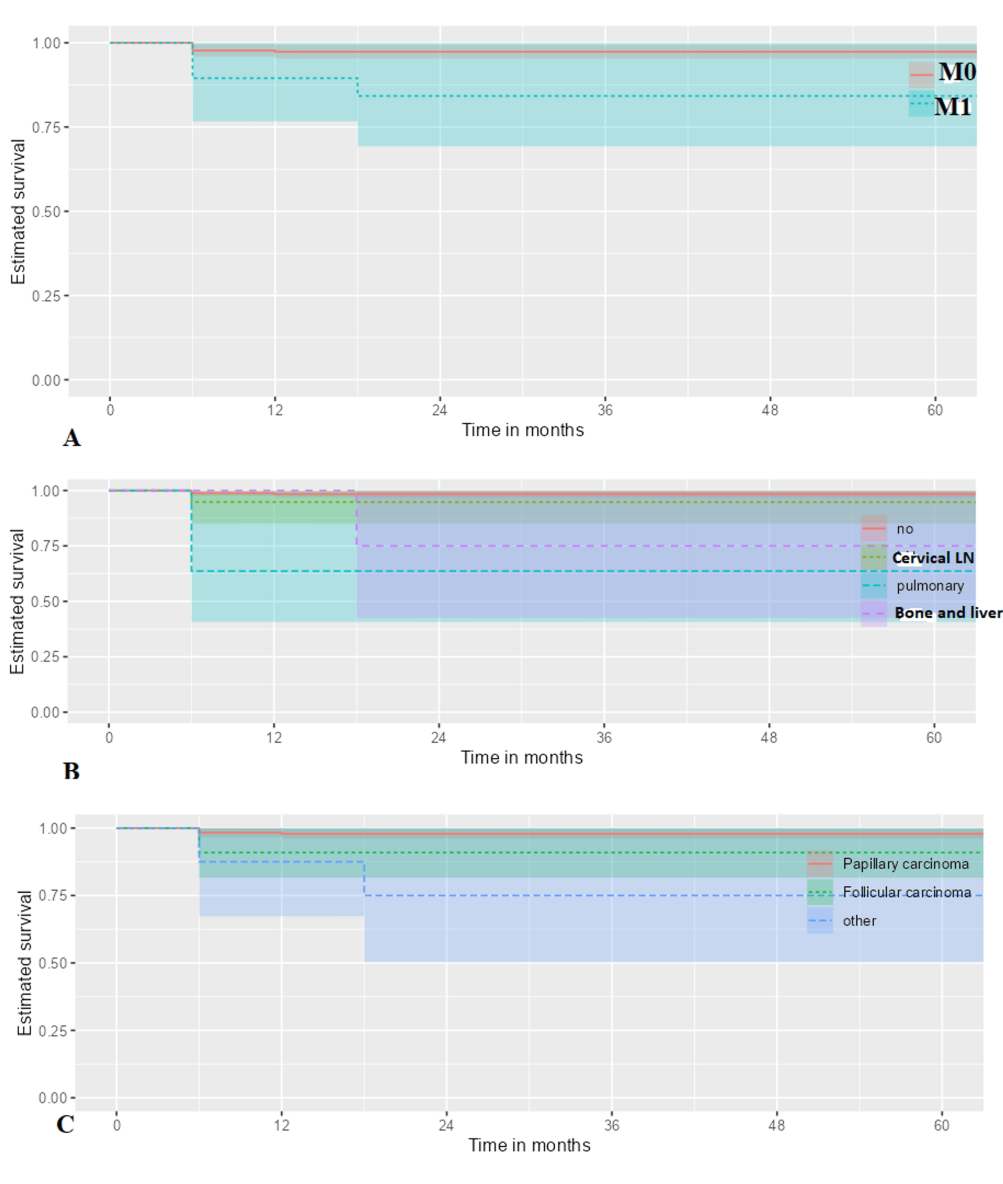
Among thyroid cancer patients, cox-proportional hazard methods found a statistically significant association between mortality and (A) distant metastasis (p<0.001), (B) metastasis locations (pulmonary metastasis; p<0.001 and bone and liver metastasis; p=0.015), (C) pathologic subtypes (follicular carcinoma (p=0.004) and other nonpapillary subtypes (p=0.002)). Cervical LN: Cervical lymph nodes

Based on univariate Cox regression analysis, independent prognostic factors of overall mortality of thyroid carcinoma were older age (HR:1.05; 95% CI: 1.01-1.09, p=0.008) (Figure 2A), diabetic mellitus (HR: 4.30; 95% CI: 1.11-16.62, p=0.035) (Figure 2B), extrathyroidal extension (HR: 6.15; 95% CI: 1.59-23.77, p=0.009) (Figure 2C), tumor size > 4 cm (HR:121.21; 95% CI: 15.33-958.34, p<0.001) (Figure 2D), metastasis presentation (HR: 11.70; 95% CI: 3.30-41.46, p<0.001) (Figure 3A), pulmonary metastasis (HR: 27.92; 95% CI: 6.96-111.98, p<0.001), bone and liver metastasis (HR: 15.20; 95% CI: 1.70-135.98, p=0.015) (Figure 3B), pathologic subtype of follicular carcinoma (HR: 4.48; 95% CI: 1.07-18.73, p=0.040) or non-papillary thyroid carcinoma subtypes (HR: 12.56; 95% CI: 2.44-64.74, p=0.002) (Figure 3C) (Table 4).

**Table 4:** Univariate Cox regression analyses of thyroid carcinoma overall survival. Abbreviations: SD, standard deviations; HR, Hazard Ratio; CI, confidence interval. Notes: * Other pathologies include anaplastic carcinoma of the thyroid, medullary thyroid carcinoma, well-differentiated thyroid tumor of uncertain malignant potential, Hurthle cell cancer, and non-Hodgkin's lymphoma. A p-value less than 0.05 was considered statistically significant.

Variable	Subgroups	N (%)	HR (95% CI)	p-value
Age (year)	Mean (SD)	43.5 (14.3)	1.05 (1.01-1.09)	0.008
Gender	Male	76 (27.2)	Reference group	0.607
	Female	203 (72.8)	1.50 (0.32-7.07)	
Hypertension	No	246 (88.2)	Reference group	0.431
	Yes	33 (11.8)	1.86 (0.40-8.78)	
Diabetic mellitus	No	253 (90.7)	Reference group	0.035
	Yes	26 (9.3)	4.30 (1.11-16.62)	
Pathology subtype	Papillary carcinoma	236 (85.2)	Reference group	
	Follicular carcinoma	33 (11.9)	4.48 (1.07-18.73)	0.040
	Other carcinomas*	8 (2.9)	12.56 (2.44-64.74)	0.002
Thyroglobulin	Normal	242 (86.7)	Reference group	0.533
	High	37 (13.3)	1.64 (0.35-7.71)	
Metastasis	No	245 (87.8)	Reference group	<0.001
	Yes	34 (12.2)	11.70 (3.30-41.46)	
Metastasis locations	No	245 (87.8)	Reference group	
	Cervical lymphadenopathy	19 (6.8)	3.30 (0.37-29.52)	0.286
	Pulmonary	11 (3.9)	27.92 (6.96-111.98)	<0.001
	Bone or liver	4 (1.4)	15.20 (1.70-135.98)	0.015
Type of surgery	Total thyroidectomy	170 (60.9)	Reference group	0.476
	Other procedures	109 (39.1)	1.57 (0.45-5.42)	
Radioactive iodine ablation	No	228 (81.7)	Reference group	0.881
	Yes	51 (18.3)	1.13 (0.24-5.30)	
Tumor size	≤ 4 cm	253 (91.7)	Reference group	<0.001
	> 4 cm	23 (8.3)	121.21 (15.33-958.34)	
Extrathyroidal extension	No	260 (93.2)	Reference group	0.009
	Yes	19 (6.8)	6.15 (1.59-23.77)	
Relapse or recurrence	No	264 (94.6)	Reference group	0.709
	Yes	15 (5.4)	1.10 (0.66-1.86)	

## Discussion

This study reviewed a single-center database of the pattern of thyroid cancer and the patients’ profile in a single cancer center. In addition, our study investigates the link between survival and prognostic factors such as comorbidities, thyroglobulin levels, treatment activity, tumor multifocality, metastasis presentation and location, residual size, and thyroidectomies subtypes in differentiated thyroid carcinoma patients.

The mean age of the patients in our study was 42.8 years, with the majority falling within the age range of 30 to 39 years (n = 85, 31%), followed by those aged 40 to 49 years (n = 73, 26%). Our findings are consistent with previous reports from Saudi Arabia, including those by Samargandy et al., Jammah et al., and Hussein et al. Furthermore, our study demonstrated a disproportionate prevalence among females, which aligns with both national and international studies [11-13]. Notably, advanced age was associated with increased mortality. Although female gender was also linked to higher mortality rates, this association did not reach statistical significance. In a study by Shah et al., age was found to be a significant predictor of treatment response, recurrence, and mortality, with younger patients exhibiting a higher percentage of favorable responses compared to older patients [14]. Jonklaas et al. further observed that prognosis varied within different age groups and between genders. Specifically, younger females (aged < 55 years) showed better outcomes, whereas females aged 55 years and older had prognoses comparable to their male counterparts [15].

Consistent with prior reports, papillary thyroid cancer accounted for the majority of the cases (84.6%) [5,11-13,16]. Follicular thyroid cancer was reported in (11.8%) of our patients. Overall, these tumors represent well-differentiated carcinomas, that carry an overall favorable prognosis [17]. Our study reveals that the pathologic subtypes of follicular thyroid carcinoma (HR: 4.48) and non-papillary thyroid carcinoma subtypes (HR: 12.56) were associated with increased mortality. These findings might be related to the tendency of follicular carcinoma to metastasize to distant organs, compared to papillary thyroid cancer which tends to metastasize to regional lymph nodes [18].

The potential association between systemic illness and thyroid cancer has been suggested, though results from various studies remain inconclusive. Elevated thyroid hormone levels, insulin resistance, obesity, and vitamin D insufficiency may all have a modest association with thyroid cancer [19]. In the present study, diabetes mellitus was observed in 9.3% of thyroid cancer patients and was significantly associated with increased mortality. However, a pooled analysis by Kitahara et al. found no significant association between diabetes mellitus or physical inactivity and thyroid malignancies [20]. Conversely, a more recent meta-analysis by Yeo et al. indicated that women with pre-existing diabetes are more likely to develop thyroid cancer compared to their non-diabetic counterparts [21]. Moreover, findings by Dong et al. demonstrated a significant link between diabetes and an increased risk of thyroid cancer in both men and women, suggesting a positive association between diabetes and thyroid cancer [19]. While these findings imply a modest correlation, further research is necessary to assess this relationship.

Total thyroidectomy was the most commonly performed surgical intervention among our patients, accounting for 60.9% of cases. Following surgery, adjuvant therapies such as radioactive iodine ablation and external beam radiotherapy were administered in 18.3% and 1.4% of patients, respectively. Notably, our approach differs from reported data by another cancer center, where radioactive iodine was utilized in the majority of cases [22]. The use of radioactive iodine is primarily dependent on the risk of recurrence and is routinely recommended by the American Thyroid Association for patients with high-risk diseases [23]. Nevertheless, risk stratification data were not reported for the patients in their study.

Long-term survival in thyroid cancer patients is influenced by surgery type and tumor size, but the prognosis is independent of prophylactic lymph node dissection in clinically node-negative disease. Previous studies have demonstrated that tumor size and lymph node involvement independently affect overall survival, regardless of the type of surgery performed [24-26]. In this study, the surgery type and lymph node dissection were unrelated to mortality. This may be due to the majority of cases (60%) undergoing total thyroidectomy and a few numbers (10.0%) undergoing total thyroidectomy with lymph node dissection. Additionally, our study discovered that pre-treatment thyroglobulin levels were connected with mortality (HR: 1.64). However, the association was not statistically significant despite earlier studies indicating a relationship between high thyroglobulin levels and disease metastasis [25,27].

In thyroid cancer, variables such as tumor size, extra-thyroidal extension, axillary lymph node status, pulmonary metastasis, histological grade, multiple organ involvement, and distant metastasis all have a substantial impact on survival, as documented in several reports [28-30]. Identifying indicators to predict and prevent organ-specific colonization in thyroid cancer patients might aid in developing follow-up measures and individualized therapy. In concordance with these findings, our study reveals that metastasis presentation, pulmonary metastasis, bone and liver metastasis, tumor size > 4 cm, and extrathyroidal extensions were associated with mortality in thyroid carcinoma patients.

In our study, the interval between the manifestation of a sign or symptom and the diagnosis of thyroid cancer varied, although, in the majority of instances, it was under three months. However, no specific details regarding this longer time to diagnosis due to the retrospective design of this study. In another report, Gianlorenzo Dionigi et al. reported that the time interval between the occurrence of a sign/symptom and thyroidectomy averaged 3 months. They found that the patient's time interval ranged from 25-85 days, diagnostic time interval from 12-40 days, and therapeutic time interval from 7-30 days. Furthermore, the patient's time interval was higher than the diagnostic time interval and therapeutic time interval and was statistically significant [31].

Study limitations

This study has several limitations. The methodological design, limited to a retrospective chart review, is subject to risks of selection and misclassification biases. Additionally, data accuracy may be compromised due to incomplete or inaccurate documentation. Moreover, the study reflects the experience of a single cancer center. Although conducted in a tertiary referral hospital, the findings may not be representative of the entire nation. Further research, involving prospective studies using a registry of consecutive cases with longer follow-up periods, is necessary to validate our results.

## Conclusions

Our study analyzed thyroid cancers in Najran between 2014 and 2021, revealing that women and individuals aged 30-39 were the most affected, and the most frequent type of thyroid cancer was papillary carcinoma. Additionally, advanced chronological age, the existence of diabetes mellitus, non-papillary thyroid carcinoma variants, metastatic progression, tumor dimensions exceeding 4 cm, and extrathyroidal extension are independently correlated with an unfavorable prognosis in individuals diagnosed with thyroid carcinoma. To provide the highest standard of contemporary medical care, a multidisciplinary framework should be utilized when formulating a customized therapeutic regimen, considering pertinent guidelines and stratification methodologies.
